# Supplementary material for: The interactome of intact mitochondria by cross-linking mass spectrometry provides evidence for coexisting respiratory supercomplexes
Source: Mol Cell Proteomics. 2017 Dec 8;17(2):216–32. doi: 10.1074/mcp.RA117.000470 (PMC5795388; doi:10.1074/mcp.RA117.000470)
Supplement: Supplemental Data [file supp_RA117.000470_133922_0_supp_23978_fzffwf.zip › spectra_annotation/mito_spectra_annotation/218-2-47-1-11-1.pdf]

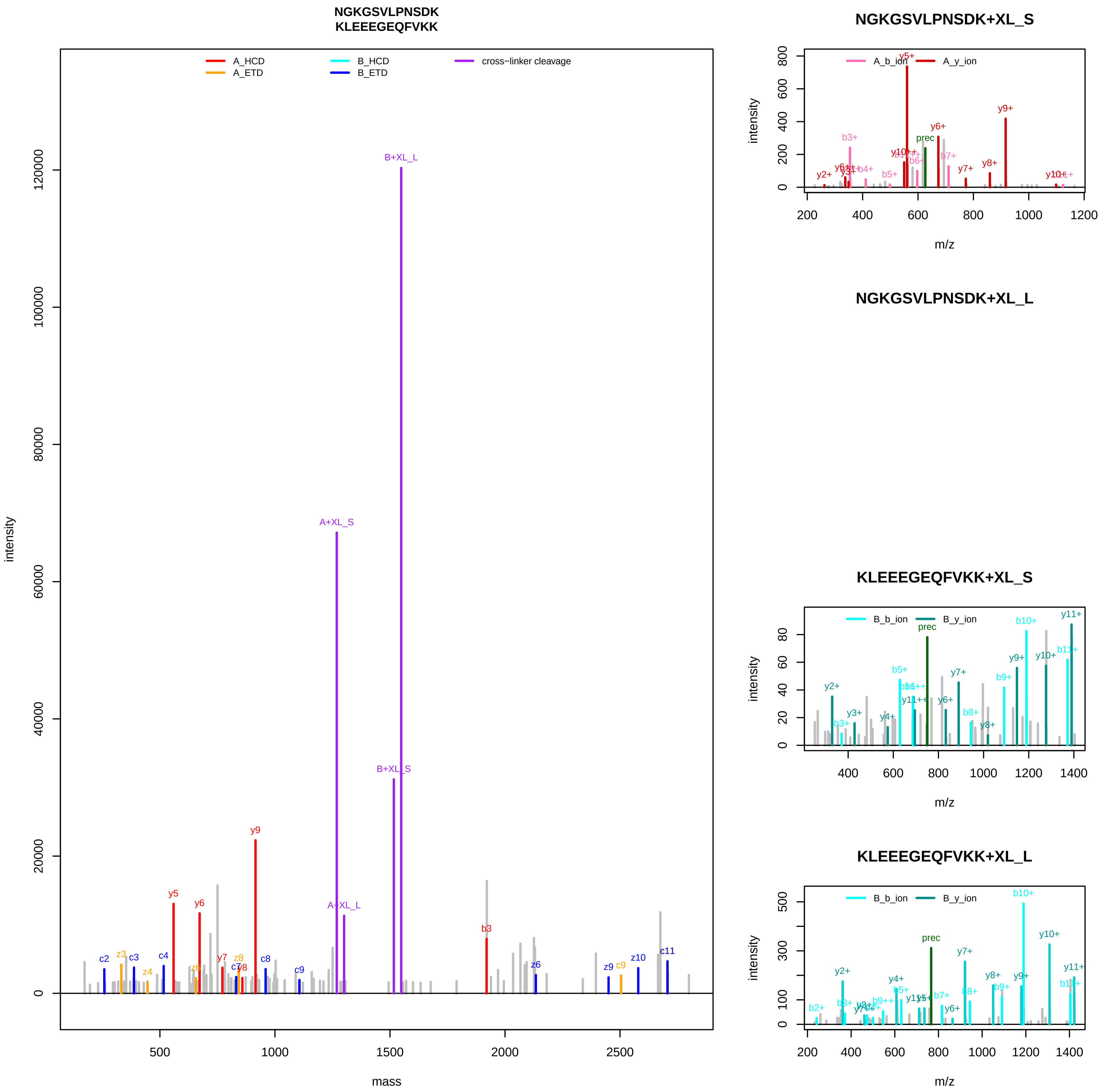
NGKGSVLPNSDK
KLEEEGEQFVKK
A_HCD
A_ETD
B_HCD
B_ETD
cross−linker cleavage
B+XL_L
A+XL_S
B+XL_S
A+XL_L
y5
y6
y7
y8
y9
b3
c2
c3
c4
c7
c8
c9
c11
z3
z4
z6
z8
z9
z10
intensity
mass
NGKGSVLPNSDK+XL_S
A_b_ion
A_y_ion
prec
y2+
y3+
y5+
y6+
y7+
y8+
y9+
y10+
b3+
b4+
b5+
b6+
b7+
m/z
NGKGSVLPNSDK+XL_L
KLEEEGEQFVKK+XL_S
B_b_ion
B_y_ion
b3+
b5+
b8+
b9+
b10+
b11+
y2+
y3+
y4+
y6+
y7+
y8+
y9+
y10+
y11+
KLEEEGEQFVKK+XL_L
b2+
b5+
b7+
b8+
b9+
